# The Effect of Bottom Profile Dimples on the Femoral Head on Wear in Metal-on-Metal Total Hip Arthroplasty

**DOI:** 10.3390/jfb12020038

**Published:** 2021-06-06

**Authors:** J. Jamari, Muhammad Imam Ammarullah, Amir Putra Md Saad, Ardiyansyah Syahrom, Mohammad Uddin, Emile van der Heide, Hasan Basri

**Affiliations:** 1 Department of Mechanical Engineering, Faculty of Engineering, Diponegoro University, Tembalang, Semarang 50275, Central Java, Indonesia; j.jamari@gmail.com (J.J.); imamammarullah@gmail.com (M.I.A.); 2 Applied Mechanics and Design, School of Mechanical Engineering, Faculty of Mechanical Engineering, Universiti Teknologi Malaysia, Skudai 81310, Johor Bahru, Malaysia; amirputra@utm.my (A.P.M.S.); ardi@utm.my (A.S.); 3 Medical Device and Technology Center (MEDiTEC), Institute of Human-Centered and Engineering (IHumEn), Universiti Teknologi Malaysia, Skudai 81310, Johor Bahru, Malaysia; 4 UniSA STEM, University of South Australia, Mawson Lakes Campus, Mawson Lakes, SA 5095, Australia; Mohammad.Uddin@unisa.edu.au; 5 Laboratory for Surface Technology and Tribology, Faculty of Engineering Technology, University of Twente, P.O. Box 217, 7500 AE Enschede, The Netherlands; e.vanderheide@utwente.nl; 6 Department of Mechanical Engineering, Faculty of Engineering, Sriwijaya University, Indralaya 30662, South Sumatera, Indonesia

**Keywords:** total hip arthroplasty, contact pressure, wear, dimple, bottom profile

## Abstract

Wear and wear-induced debris is a significant factor in causing failure in implants. Reducing contact pressure by using a textured surface between the femoral head and acetabular cup is crucial to improving the implant’s life. This study presented the effect of surface texturing as dimples on the wear evolution of total hip arthroplasty. It was implemented by developing finite element analysis from the prediction model without dimples and with bottom profile dimples of flat, drill, and ball types. Simulations were carried out by performing 3D physiological loading of the hip joint under normal walking conditions. A geometry update was initiated based on the patient’s daily routine activities. Our results showed that the addition of dimples reduced contact pressure and wear. The bottom profile dimples of the ball type had the best ability to reduce wear relative to the other types, reducing cumulative linear wear by 24.3% and cumulative volumetric wear by 31% compared to no dimples. The findings demonstrated that surface texturing with appropriate dimple bottom geometry on a bearing surface is able to extend the lifetime of hip implants.

## 1. Introduction

Metal-on-metal is one of the available bearing combination options for total hip replacement surgery that has better stability, resulting in fewer dislocations. It has good hardness compared to ceramic materials, resulting in a lower rate of fracture failure under high loads, and wear rates 20–100 times lower than conventional metal-on-polyethylene hip joint bearings [1]. This type of bearing can also be an alternative for younger and more active patients [2]. Although metallic materials are not the best choice compared to other material combinations due to the relatively high number of failure cases requiring revision operations [3], these materials are still used in several developing countries, including in Indonesia, because of their affordable price and availability of production equipment, which are required in order to meet national market demand independently, without imports [4]. Under the expected conditions, the metal-on-metal wear level is low, at less than 0.3 mm^3^/10^6^ cycles, though it will produce microparticles [5]. The main drawback of this type of bearing is that direct contact with metal-on-metal can release metal ions into the bloodstream that then spread throughout the body, creating local inflammation, a reaction that ultimately contributes to the emergence of osteolysis. Worn metal particles can spread through the lymphatic system to locations far from the implant, and it has been reported that the metals can accumulate in the liver, spleen, lymph nodes, and bone marrow. Along with the reactive nature of metal wear particles, they have the potential to cause cytotoxicity, hypersensitivity, and neoplasia. Minimizing wear in metal-on-metal bearings is very important in order to avoid the risk of poisoning [6,7].

Textured surface applications are widely applied to various mechanical components. Total hip arthroplasty with a textured surface can reduce the surface contact area, and decrease the adhesion wear and coefficient of friction [8]. Dimples serve to trap wear particles, thereby preventing abrasive wear of the contact surface by a third object, and generating hydrodynamic pressure to provide additional lift [9,10]. Various studies have also shown the positive effect of surface texturing on bearings in improving the tribological performance, both theoretically and experimentally [11,12,13,14].

Some parameters that influence adding dimples to reduce wear in total hip arthroplasty, such as shape, diameter, depth, distance, direction, and arrangement, have been investigated previously in order to maximize the effect of adding dimples, and to find the optimal parameters. Even so, until now, there has been no research to determine the optimum conditions for various parameters; these are usually investigated by trial and error [9,10]. One of the relatively new parameters being studied by many researchers is the bottom profile geometry of dimples. Pratap and Patra [13] studied micro dimples, whose bottom profiles were varied with flat, drill, and ball types to increase the wettability experimentally by using the pin-on-disk method, and reported that the ball type bottom profile produces smoother wear patterns and the lowest wear coefficient compared to other bottom profiles. Research related to bottom profile parameters was also carried out by Wang et al. [12] on axial bearings with variations in the bottom profile dimple groove type, to simulate the fluid flow characteristics using the computational fluid dynamic method. It has been explained that different bottom profiles impact the overall axial bearing performance by increasing the hydrodynamic pressure and minimum film thickness. However, until now, research related to bottom profile dimple parameters in total hip arthroplasty has never been carried out, and should be explored further.

The finite element method, as a computational analysis tool, has been widely applied to various total hip arthroplasty studies, minimizing the need for costly experiments. To shorten this time-consuming computational process, many studies, such as those by Cosmic et al. [15], Liu et al. [16], Jamari et al. [17], Harun et al. [2], Meng et al. [6], and Basri et al. [9,10], have used simplified loading, with vertical loads that did not represent the true physiological hip joint. This may limit the actual results, including analysis of the effect of adding dimples on wear, which needs to consider gait loading and the range of motion of the hip joint in actual conditions. Contact pressure on the bearing surface is significant in terms of wear, and therefore significantly affects the survival of the total hip arthroplasty; a higher contact pressure leads to higher wear [2,6,18,19,20]. Therefore, reducing contact pressure, especially in high gait loading areas, is a strategic step that can be taken to reduce wear and extend the implant’s life.

Total hip arthroplasty wear modelling by previous researchers has mostly used synovial fluids as lubrication, to assess the actual conditions [6,9,10]. Dry contact, which reflects boundary lubrication conditions, is also essential, and contact pressure can be seen more clearly under dry contact conditions. The contact pressure is one of the mechanical parameters of contact analysis associated with contact surface damage and wear [6]. Previous modelling of dry contact has been published by Pedersen et al. [21], Meng et al. [6], Jamari et al. [17], Uddin and Zhang [18], Harun et al. [6], Shankar et al. [19], Nithyaprakash et al. [20], and Cosmi et al. [15]. Dry contact wear studies with a textured surface on a hip joint prosthesis, to assess this surface under boundary lubrication conditions, have not provided a complete understanding as yet.

To address this problem, this study investigated the effects of bottom profile dimples on wear in hip implants. A 3D finite element model, consisting of a metallic acetabular cup surface and a metallic femoral head with dimples, was developed. Gait loading and range of motion in 3D were presented according to the hip joint’s physiological conditions, to provide realistic simulation conditions. A geometry update was carried out to reflect changes in worn surface features due to wear.

## 2. Materials and Methods

### 2.1. Geometric Parameters and Material Properties

For the geometric parameters commonly used for bearing couples in total hip arthroplasty, including both the acetabular cup and femoral head, we refer to previous studies conducted by Mak et al. [22], described in Table 1. 

Textured surface applications were performed by adding dimples to the femoral head contact surface, referring to previous studies conducted by Pratap and Patra [13] and Choudhury et al. [14], and explained in Table 2.

Metal-on-metal bearings are made of a cobalt chromium molybdenum material, assumed to be homogeneous and isotropic, for the femoral head and acetabular cup components. Material properties required for the simulation process included Young’s modulus, Poisson’s ratio, and density, as described in Table 3. The coefficient of friction used in this study for the untextured model was 0.2 [18], and that for the textured model was 0.16 [14].

### 2.2. Finite Element Modelling

**Figure 1:**
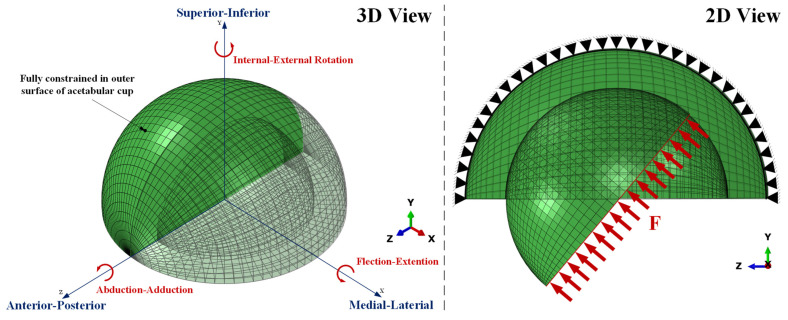
Finite element contact model of the femoral head and acetabular cup.

The two main components of the hip joint implant in the wear simulation were the acetabular cup and femoral head. We defined the contacting acetabular cup surface as the master surface and the contacting femoral head surface as the slave surface. To minimize computational complexity and analysis time, with results that would be close to actual conditions, we used a ball-in-socket 3D model with an asymmetric femoral head; the stem section of the femoral head was not considered [11,18,19,20]. Barreto et al. [21] reported that considering the pelvic bone in the computational model does not significantly affect the results of contact pressure. Therefore, this study did not consider the pelvic bone, in order to shorten the required simulation time. Micro-separation of the femoral head against the acetabular cup was not allowed, and it was positioned concentrically. A mesh convergence study was performed for models without and with flat, drill, and ball bottom profile dimples. The optimal number of elements for each model was 44,200, 892,427, 885,251, and 887,192, respectively, using 41,600 C3D8 and 2600 C3D6 for without dimples, then 3840 C3D8, 256 C3D6, and C3D10 for the dimple model. Figure 1 shows various parameters applied to finite element analysis through numerical analysis to obtain the desired solution. The steady-state contact mechanic was applied using ABAQUS/CAE 6.14-1 to simulate contact, where the material defined in this simulation was assumed to be linear elastic. In order to represent lubrication and surface roughness against progress of wear, this study considered the presence of friction between surfaces. Temperature changes during contact were considered constant. Additionally, the acetabular cup was made unable to move and was given a maximum range of motion in the femoral head.

**Figure 2:**
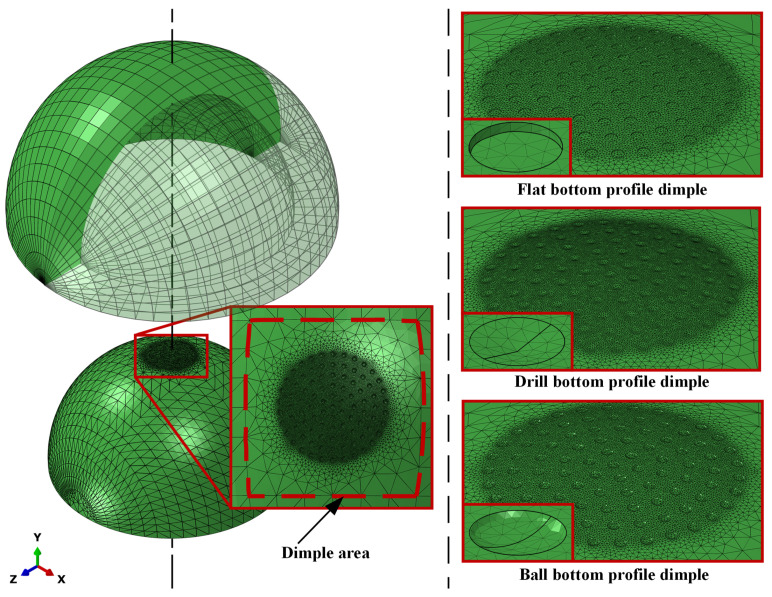
Meshing on the dimple area.

In calculating wear based on contact nodes between surfaces, elements must produce nodes that meet each other. We adopted the duel-pole meshing method, as reported in Pedersen et al. [21] Fine meshing was performed in the dimple area, as shown in Figure 2. Since the bearing in contact experienced varying contact pressure, reducing its contact pressure, especially in areas with the highest contact pressure distribution area, is a strategic step to reduce wear. Many previous studies have shown that the highest contact pressure is in the center area of the bearing [2,18,19,20]; therefore, a textured surface application was developed, based on the highest contact area, consisting of 91 dimples with different bottom profiles, and added to the femoral head dimple parameters from Pratap and Patra [13] and Choudhury et al. [14]

### 2.3. Gait Cycle

**Figure 3:**
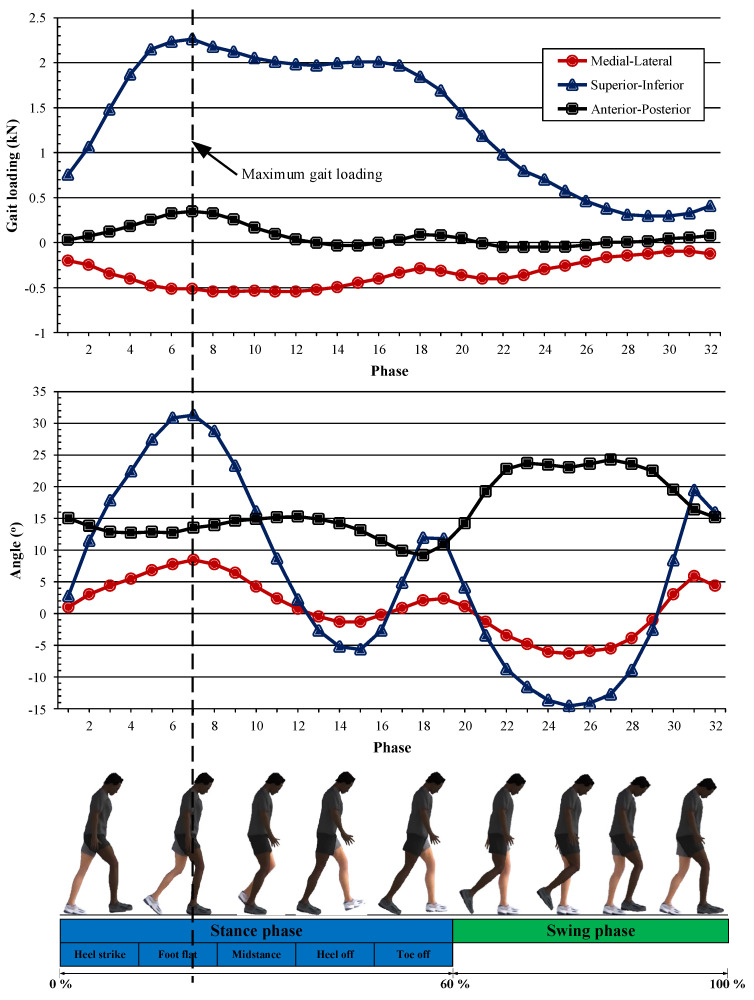
Gait cycle under normal walking conditions [11].

In general, the human activity most commonly performed in the daily life of patients who have undergone a total hip replacement surgery is normal walking. Through rationalization, a recent study provided normal walking conditions in a simulation. To obtain a walking cycle approach under normal walking conditions with physiologically representative 3D hip joints, we referred to Bergmann et al.’s results for hip loading under normal walking conditions [23], as presented in Figure 3. These data were obtained from an experimental analysis of gait loading measurements of a left-sided hip joint following total hip arthroplasty, based on the average of four different users, described by 200 instances. To simplify the calculation, this cycle was divided into 32 phases [18,19,20]. The first 1–19 phases were called the ‘stance phase’ (the first 60% of the cycle), and the next 20–32 phases were called the ‘swing phase’ (the last 40% of the cycle). The magnitude and direction of the load acting on the hip joint varied depending on the phase changes of the normal walking cycle; the maximum hip joint load was 2326 N (around 2.5–3 times the average human body weight), occurring at the 7th phase of motion, at the peak of the stance phase.

### 2.4. Wear Model

In calculating the wear, we adopted Archard’s wear law [24] in the form of abrasive-adhesive wear at a point *P* (node) from the bearing surface, and at time *t*, as in Equation (1):

(1) $$W_{V}\left(P,t\right)=K_{w}\left(P,t\right)F\left(P,t\right)s\left(P,t\right)$$

where *W_v_* is the volumetric wear, *K_w_* is the wear coefficient obtained from the hip joint simulator or pin-on-disc study, *F* is the contact force obtained from the measurement of the hip joint force, and *s* is the sliding distance obtained from movement of the femoral head in 3D on the acetabular cup. This equation can be modified by dividing the two segments by the contact area that occurs, so that it becomes Equation (2):

(2) $$W_{L}\left(P,t\right)=K_{w}\left(P,t\right)P\left(P,t\right)s\left(P,t\right)$$

*W_L_* is the linear wear and *P* is the contact pressure obtained from finite element analysis.

### 2.5. Wear and Geometry Update

Wear changes the bearing surface geometry, affecting the contact pressure. The surface geometry is updated by moving nodes in the radial direction by the amount of linear wear, then surfaces with renewed geometry are remeshed. Ideally, geometry updates should occur every time a gait cycle is completed. However, this would take a long time and would be very ineffective in simulation studies. In various studies, the hip joint’s average normal walking activity has been found to be 1 × 10^6^ cycles/year [18,19,20]. To provide clear results for investigating the cumulative wear trend of linear and volumetric wear, this study estimated two-year wear, equivalent to 2 × 10^6^ cycles/two years, as was widely reported by previous studies [18,19]. Geometry updates were performed every 2 × 10^5^ cycles, so that during a wear prediction period of 1 year, the geometry would be updated five times. This was seen as sufficient to calculate the change in contact stress due to wear accurately. The wear coefficient affects the overall wear, and some types of couple bearings have a constant wear coefficient, but for metal-on-metal, the wear condition is divided into two phases: running-in and steady-state, whose values were based on the hip joint simulator study reported by Chan et al. [25], and described in Table 4. The running-in phase was taken in the first year, and continued for the steady-state phase taken in the following year.

## 3. Results and Discussion

### 3.1. Contact Pressure Validation

**Figure 4:**
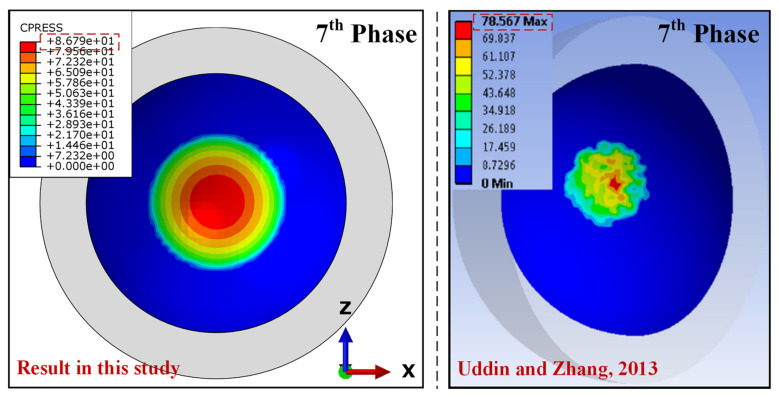
Contact pressure validation after 2 × 10^6^ cycles for the hip model without dimples.

Contact pressure was validated based on research conducted by Uddin and Zhang [18] for the without-dimple model, where they obtained the highest value of contact pressure in the 7th phase (67.74 MPa) in the initial cycle, and this increased to 78.56 MPa after 2 × 10^6^ cycles. Meanwhile, for the current research, the highest contact pressure in the 7th phase was 78.2 MPa in the initial cycle, and this increased to 86.79 MPa. From the comparison between the results of the current study and the study by Uddin and Zhang shown in Figure 4, it can be said that the results of this study were slightly higher, but still acceptable, because the difference was insignificant. Further discussion regarding the results of contact pressure will be explained in the next section.

### 3.2. Contact Pressure Analysis

**Figure 5:**
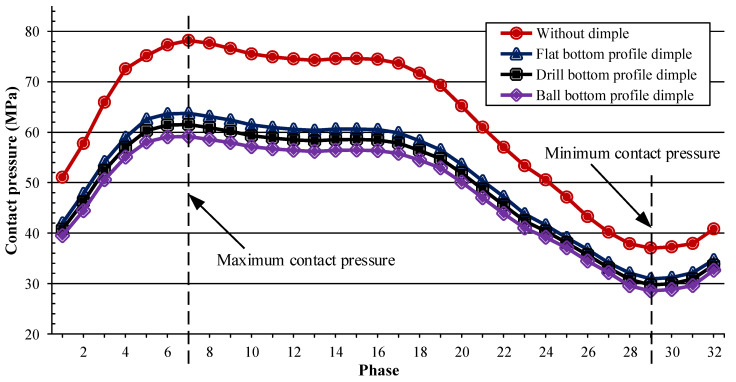
Contact pressure on acetabular cup surface at the initial cycle.

One of the indicators determining the calculation of wear using Archard’s wear equation is contact pressure [24]. Before further carrying out the wear analysis, contact pressure analysis was conducted in the initial cycle to obtain a contact pressure comparison between the models used in the study; this comparison is shown in Figure 5. We observed a correlation of contact pressure magnitude with cumulative linear wear and cumulative volumetric wear. The results obtained were rational, because in the partition of 32 phases for one normal loading cycle [18,19,20], the highest contact pressure was in the 7th phase, which was part of the standing phase. If we look at the physiological conditions of the hip joint, the 7th phase is a condition wherein the hip joint supports the entire body weight to move the other leg, while the other leg experiences a swinging phase [23]. The lowest contact pressure occurred in the 29th phase, which was part of the swing phase. It should be emphasized that this study only considered contact pressure, along with the wear calculation, in dry conditions and on acetabular cup, as in the research by Uddin and Zhang [18], Shankar et al. [19], and Nithyaprakash et al. [20]

Variations in the bottom profile dimples affect contact pressure, and this will affect wear. This finding is very important, and needs further investigation. The maximum contact pressure value for all models was in the 7th phase, with a value of 78.2 MPa for the model without dimples, and 63.74 MPa, 61.54 MPa, and 59.17 MPa for the bottom profile dimple variations of flat, drill, and ball type, respectively. For the same models, the minimum contact pressures at the 29th phase were 37.04 MPa, 30.89 MPa, 29.71 MPa, and 28.53 MPa, respectively. It can be seen that ball type bottom profile has the lowest contact pressure, and is therefore expected to exhibit the lowest wear.

**Figure 6:**
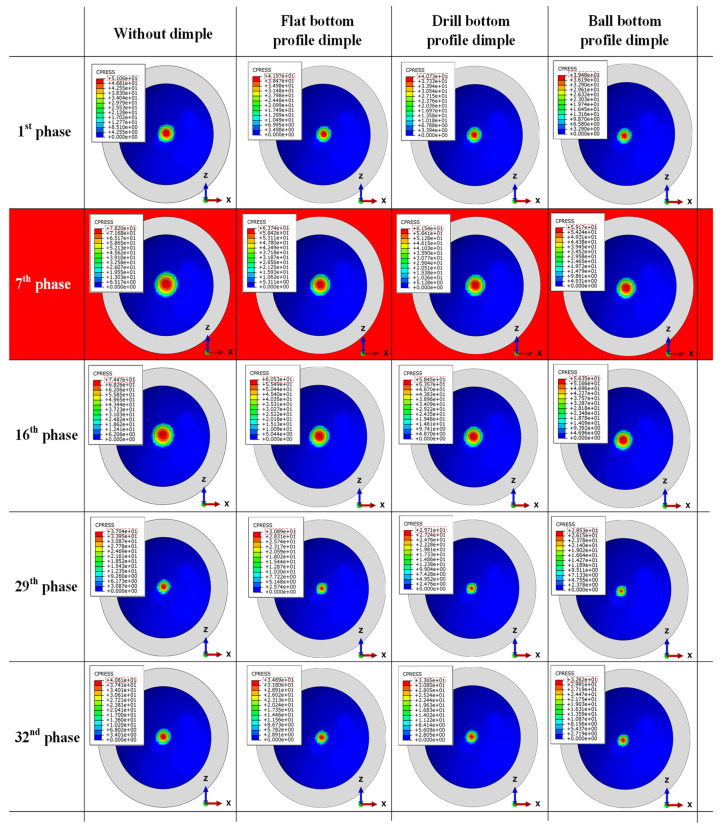
Distribution of the contact pressure on the acetabular cup surface in specific phases at the initial cycle.

Figure 6 shows the distribution of contact pressure on the acetabular cup surface during the initial cycle for all models. Contact is always around the center of acetabular cup surface. This is because current research considers the ideal normal gait. However, under actual conditions, strip wear can occur due to micro separation or other unexpected activities. This scheme created high concentration loads on the edge of the acetabular cup because of an abnormal gait. Additionally, it led to strip wear on the acetabular cup [22]. Dislocation of the femoral head relative to the acetabular cup may occur in this case. Contact pressure plots of the dimple models decreased slightly with a similar pattern, compared with the without dimple model. However, there were no significant changes for different bottom profile models because the contact surface was the same, but with a different contact pressure magnitude.

### 3.3. Wear Validation

**Figure 7:**
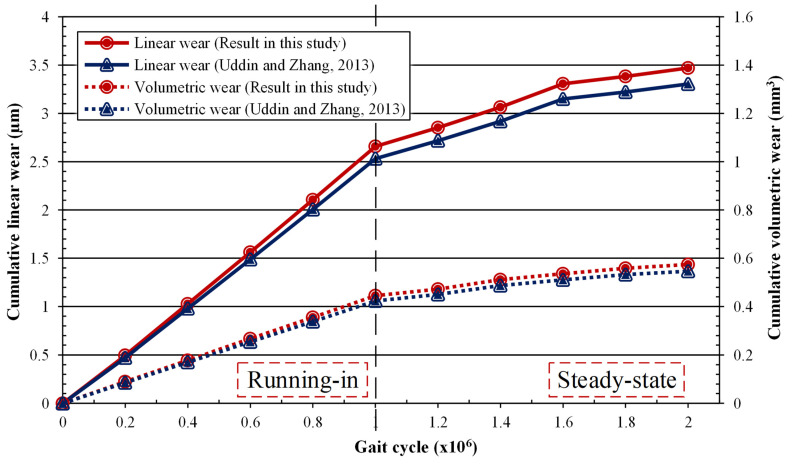
Wear validation for the hip model without dimples.

Before further analysis was conducted regarding linear and volumetric wear evaluation, wear validation was carried out to confirm the results obtained through previous research. Uddin and Zhang’s research [18] was used as validation of the without-dimple model described in Figure 7. The wear obtained in this study was higher compared to Uddin and Zhang’s findings, because the contact pressure magnitude obtained was also higher.

### 3.4. Wear Evaluation

**Figure 8:**
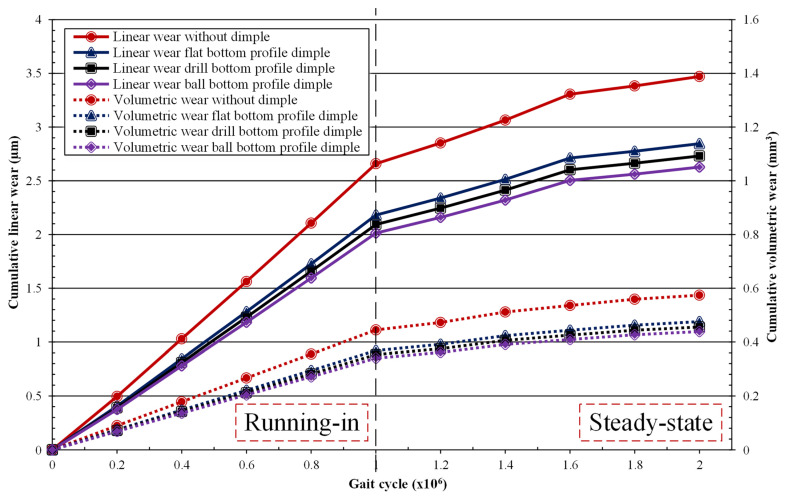
Cumulative wear on the acetabular cup surface.

Figure 8 shows the cumulative linear wear and cumulative volumetric wear for the without- and with-dimple models. It can be seen that the wear rates for all models were very high in the first 1 × 10^6^ cycles, but decreased at the second 1 × 10^6^ cycles. This was due to the relatively high wear coefficient on the running-in phase compared to the steady-state phase [25]. For the without-dimple model, the linear wear rate for first 1 × 10^6^ cycles was 2.659 μm, but this then decreased to 0.81 μm at the second 1 × 10^6^ cycles. From these results, the prediction of wear for the without-dimple model was in accordance with the measurements from laboratory studies by Medley et al. [26] at around 1.27 to 15.7 µm, as well as the clinical testing from Reinisch et al. [27] at around 2.9 to 12.8 μm in the first 1 × 10^6^ cycles.

Regarding the volumetric wear rate, for the model without dimples for the first 1 × 10^6^ cycles, it was 0.444 mm^3^, and for the second 1 × 10^6^ cycles this changed to 0.12 mm^3^. The results obtained were similar to wear testing results using a hip joint simulator found by Chan et al. [25], where the wear rate for the first 1 × 10^6^ cycles was 0.22 mm^3^ and for the rest it was 0.065 mm^3^/1 × 10^6^ cycle. In addition, clinical testing data from Reinisch et al.’s work [27] explained that the mean of the volumetric wear rate for each 1 × 10^6^ cycles was 3.74 mm^3^. Comparing the simulation results with simulations carried out in the previous section, the laboratory and clinical tests were also vital to ensure the accuracy of the results. 

The cumulative linear wear values after 2 × 10^6^ cycles for models without dimples and with bottom profile dimple types of flat, drill, and ball were 3.47 μm, 2.84 μm, 2.73 μm, and 2.63 μm, respectively. Meanwhile, the volumetric wear values of the same models were 0.57 mm^3^, 0.48 mm^3^, 0.45 mm^3^, and 0.44 mm^3^, respectively. Through the data presented, it is clear that dimple addition can reduce wear—both linear and volumetric. Compared with the model without dimples, the flat bottom profile dimples can reduce linear and volumetric wear by 18% and 17.2%, the drill type by 21.3% and 20.6%, and the ball type by 24.3% and 23.6%. The ball type achieved the best performance in reducing wear compared to the other models from this data presentation. These findings are in line with Pratap and Patra [13], who explained that ball bottom profile dimples produce a smoother wear pattern and the lowest wear coefficient when compared to flat, drill, and without-dimple models for the Ti-6Al-4V alloy. Our results show, under dry contact, that lower wear was found in the textured models compared to the non-textured model. This is because the coefficient of friction is reduced for textured models compared to non-textured models, enhancing the wear performance.

It should be understood that the prediction of linear and volumetric wear rates can give lower or higher results through laboratory or clinical studies. For linear wear measurements, this could be due to wear coefficients being lower or higher than those estimated by the pin-on disc or hip joint simulator [28]. For volumetric wear measurements, the inherent properties of the techniques/methods used, such as radiographic imaging techniques and computational processing in clinical studies, can significantly influence wear volume measurement, resulting in unpredictable result variations [29]. The effect of accuracy and the type of tool, user error, inaccuracy in technique/method/procedure, surrounding environment, or other factors can affect the experimental wear measurement results. Therefore, wear rates obtained through laboratory and clinical studies can vary over a wide range due to various uncertain aspects, making it challenging to present a full and in-depth understanding of wear mechanisms.

### 3.5. Change in Contact Pressure due to Wear

**Figure 9:**
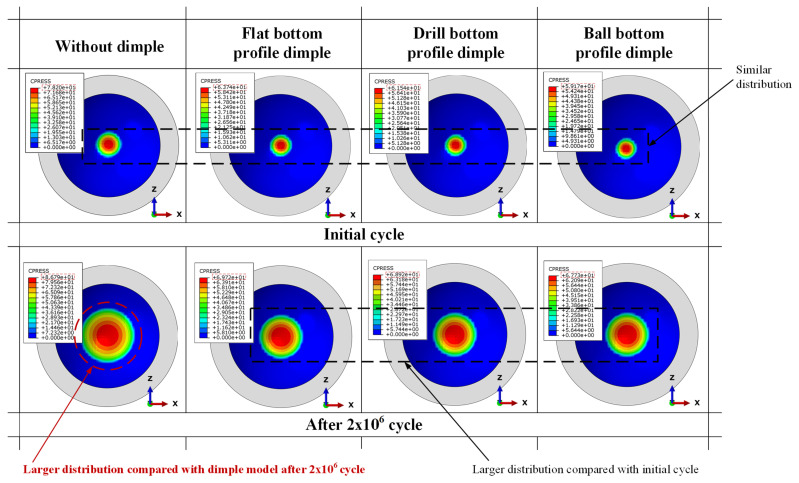
Distribution of the contact pressure on the acetabular cup surface in the 7th phase at the initial cycle and after 2 × 10^6^ cycles.

Figure 9 describes the change in contact pressure distribution on the acetabular cup surface in the 7th phase (at maximum load) during the initial cycle and after 2 × 10^6^ cycles. As the gait cycle increases, the contact pressure will be more evenly distributed on the acetabular cup surface due to local wear. An increase in contact pressure occurred for all models between the initial cycle and after 2 × 10^6^ cycles, wherein the without-dimple model increased from 78.2 MPa to 86.79 MPa (an increase of 8.59 MPa). The ball type bottom profile dimple model increased from 63.74 MPa to 69.72 MPa (an increase of 5.98 MPa), the drill bottom increased from 61.54 MPa to 68.92 MPa (an increase of 7.38 MPa), and the ball model increased from 59.17 MPa to 67.73 MPa (an increase of 8.56 MPa). Furthermore, as shown in Figure 9, the textured surface can withstand local wear, as evidenced by the distribution of the contact pressure after 2 × 10^6^ cycles, which was not too wide compared to the model without dimples. The explanation for the results presented is consistent with the available literature, which suggests various positive effects of textured surface applications on contact surfaces [9,10,12,13,14].

However, as the gait cycle increases over a long period of time, after more than about 15 × 10^6^ cycles (equivalent to 15 years of implant use), it is estimated that the maximum contact pressure will decrease as the bearing surface becomes fitter and smoother due to ongoing local wear, because the radial distance is reduced. This will cause an increase in the contact area, impacting decreases in wear progressivity, in line with the findings of Harun et al. [2]. Long-term use of dimples is expected to provide a more significant difference in results than without-dimples, because it cumulatively reduces local wear as cycles progress, ultimately showing a significant difference in wear rates compared to without-dimples over prolonged implant use.

Wear is the main cause of metal-on-metal implant failure [2,6,9,10,18,19,20,30]. Therefore, reducing wear is a strategic step in minimizing harmful failures for users. Metal-on-metal bearings also carry the danger of poisoning due to metallosis, because of metal ions entering the body from metal wear particles [9,10,30]. The results and discussion presented here show that dimples can reduce the contact pressure and wear, indicating that in real use it could reduce failure due to wear and poisoning due to metallosis. This study shows that the bottom profile dimple ball type is estimated to produce the lowest wear compared to other models; this model demonstrated a promising surface textured parameter, and could be used to design the bearing components in a total hip arthroplasty. It is necessary to consider the cost of making micro-textures, as presented in the wear models; in real life this is not cheap or easy, and the tool readiness for fabricating textured surfaces precisely needs to be considered further.

There are several limitations in this study that might have impacted the wear prediction. First, the use of constant wear coefficients during the simulation, both for models without dimples or with variations in bottom profile dimples, may have impacted the results. In the actual conditions when the wear occurs, contact between the acetabular cup and the femoral head will change in geometry and affect lubrication. Ideally, the lubrication effect is taken into account by changing the wear coefficient. Regarding lubrication, based on the investigation of Basri et al. [1,2] and supported by Pratap and Patra [8] and Wang et al. [7] who described the influence of a textured surface on lubrication performance, the variation of the bottom profile dimples has a crucial influence on lubrication. However, lubrication was not considered in this study. As for the wear coefficient, this study also used a constant coefficient of friction. In fact, the results of Choudhury et al. [5] showed that the coefficient of friction changes over time, and the application of textured surfaces also affects this coefficient, and its value changes during implant use. Lastly, the current wear model was based solely on abrasion–adhesion wear, which does not reflect other wear mechanisms such as fatigue, corrosion, and erosion, which are important in wear models. The shortcomings of the current research will need to be addressed in the future.

## 4. Conclusions

This research contributes to the development of textured surfaces in total hip arthroplasty to reduce contact pressure and wear. It uses a dimple model with a bottom profile of the flat, drill, and ball types. From this comprehensive study, the following conclusions were found: the maximum contact pressure in the acetabular cup changes with increasing gait cycles, with the highest in the 7th phase (in the standing phase), corresponding to a normal gait at the highest load, and the lowest at the 29th phase, in the swing phase. The ball bottom profile dimple model performed best at reducing contact pressure and wear on the acetabular cup compared to other models, reducing cumulative linear wear by 24.3% and cumulative volumetric wear by 23.6% compared to the without-dimple model. Textured models also have a lower coefficient of friction, which enhances the wear performance. The results also suggested that the presented simulation models in this study can be used to provide a reasonable estimate of the evolution of wear on hip joint prosthetics.

## Figures and Tables

**Table 1:** Geometric parameters of the bearing couple.

Parameter	Size
Femoral head diameter	28 mm
Radial clearance	50 μm
Acetabular cup thickness	5 mm

**Table 2:** Dimple addition parameters for the femoral head surface contact.

Parameter	Size
Diameter	0.26 mm
Depth	0.03 mm
Shape	Circle
Pattern	Circular
Pattern number	6
Dimple number	91
Pitch	0.489 mm
Addition area	Femoral head surface
Variation	Bottom profile (flat, drill, and ball)

**Table 3:** Material properties of cobalt chromium molybdenum.

Parameter	Size
Young modulus (E)	210 GPA
Poisson ratio (υ)	0.3
Density (ρ)	8300 kg/m^3^

**Table 4:** Wear coefficient of the couple bearing.

Condition	Wear coefficient (mm^3^/Nmm)
Running-in	5 × 10^−12^
Steady-state	1.5 × 10^−12^

## Data Availability

The data presented in this study are available on request from the corresponding author.
